# Over 20 years of chronic patellar ligament rupture with severe knee osteoarthritis for Total knee Arthroplasty: a case report

**DOI:** 10.1186/s12891-020-03374-3

**Published:** 2020-06-03

**Authors:** Xuejun Zhang, Yun Li, Jianfeng Chen, Chao Yan, Xiaoyi Tan, Huafang Liu

**Affiliations:** 1 Department of Orthopedics, The People’s Hospital of China Three Gorges University, First People’s Hospital of Yichang, No.4 Hudi Street, Yichang, 443000 Hubei Province China; 2 Department of Nephrology, The Jianli people’s hospital, No.55 Jiang Cheng Road, Jianli County, Jingzhou, 430022 Hubei Province China

**Keywords:** Osteoarthritis, TKA, Chronic patellar ligament rupture

## Abstract

**Background:**

Extensor apparatus rupture is a severe complication after knee arthroplasty, but there have not been many reports on how to perform knee arthroplasty after chronic patellar ligament rupture. We reported a case of total knee arthroplasty (TKA) in a patient with severe osteoarthritis (OA) complicated by chronic patellar ligament rupture.

**Case presentation:**

In this case, a 67-year-old male patient suffered from patellar ligament rupture due to trauma more than 20 years ago and did not undergo any formal treatment. Physical examination revealed a small amount of fluid and extension lag, and the patella was displaced upward by approximately 5.5 cm. The quadriceps were atrophic and weak. There was significant tenderness on the medial side of the left knee joint. Passive motion of the left knee joint ranged from full extension to 120° of flexion with discomfort during excessive flexion. Active flexion of the knee joint to 120°, and extensor lag was approximately 90°. We reconstructed the extensor apparatus through a quadriceps tendon V-Y quadricepsplasty and Krackow suture technique of the patellar ligament, and osteoarthritis was resolved with TKA. The visual analogue scale (VAS) score decreased from 5 points to 1 point after surgery. Six weeks later, the patient was able to walk normally without a walking stick, and the knee joint could stretch actively to approximately 30°. However, he had obvious extension lag. This problem improved 10 months after surgery. The AKS score increased from 35 to 95 10 months after surgery. The HSS score increased from 43 to 93.

**Conclusions:**

TKA and ligament reconstruction are options for the treatment of knee OA with chronic patellar ligament rupture. V-Y lengthening of the quadriceps femoris tendon after the Krackow suture technique of the patellar ligament with transpatellar tunnels may be a reasonable choice during TKA.

## Background

The extensor apparatus includes the quadriceps, patella, and patellar ligament. The function of the extensor apparatus is to actively extend the knee, stabilize the knee joint, and strengthen the knee joint capsule. Rupture of any of these structures can cause active knee extension lag and disabling functional consequences. Patellar ligament rupture is considered a low-frequency disease. Some patients are not able to receive corrective treatment at an early stage. Early treatment is critical for the prognosis of patients. After the patellar ligament is broken, the patella is displaced upwards under traction of the quadriceps tendon, resulting in dislocation of the patella. It is difficult for the patella to descend due to chronic patellar ligament rupture. Bonnin et al. summarized several strategies for reconstructing the patellar tendon: simple repair, repair with augmentation by a neighbouring tendon, reconstruction using an artificial ligament, replacement with an allograft and salvage techniques. End-to-end suturing becomes very difficult 45 days after injury [1]. We can use the semitendinosus band [1, 2], an artificial ligament, an allograft [3], or resorbable material [4] to strengthen the patellar ligament.

OA is an essential reason for surgical treatment of the knee joint. The absolute contraindications for knee arthroplasty include infection, an incomplete knee extensor mechanism or severe dysfunction, recurvatum deformity secondary to neuromuscular weakness, and the presence of a painless, well-functioning knee arthrodesis [5]. According to these standards, OA with chronic patellar ligament rupture might be an absolute contraindication for TKA. In most clinical guidelines, there is no absolute surgical contraindication except for infection. We can perform knee extensor mechanism reconstruction while performing TKA at the same time, for extensor tendon rupture after TKA. In this article, we report a case of TKA in a patient by knee osteoarthritis complicated with chronic patellar ligament rupture. We developed a new method to perform TKA and ligament reconstruction. V-Y lengthening of the quadriceps femoris tendon after the Krackow suture technique of the patellar ligament with transpatellar tunnels may be an excellent choice to reconstruct the patellar ligament.

## Case presentation

**Fig. 1:**
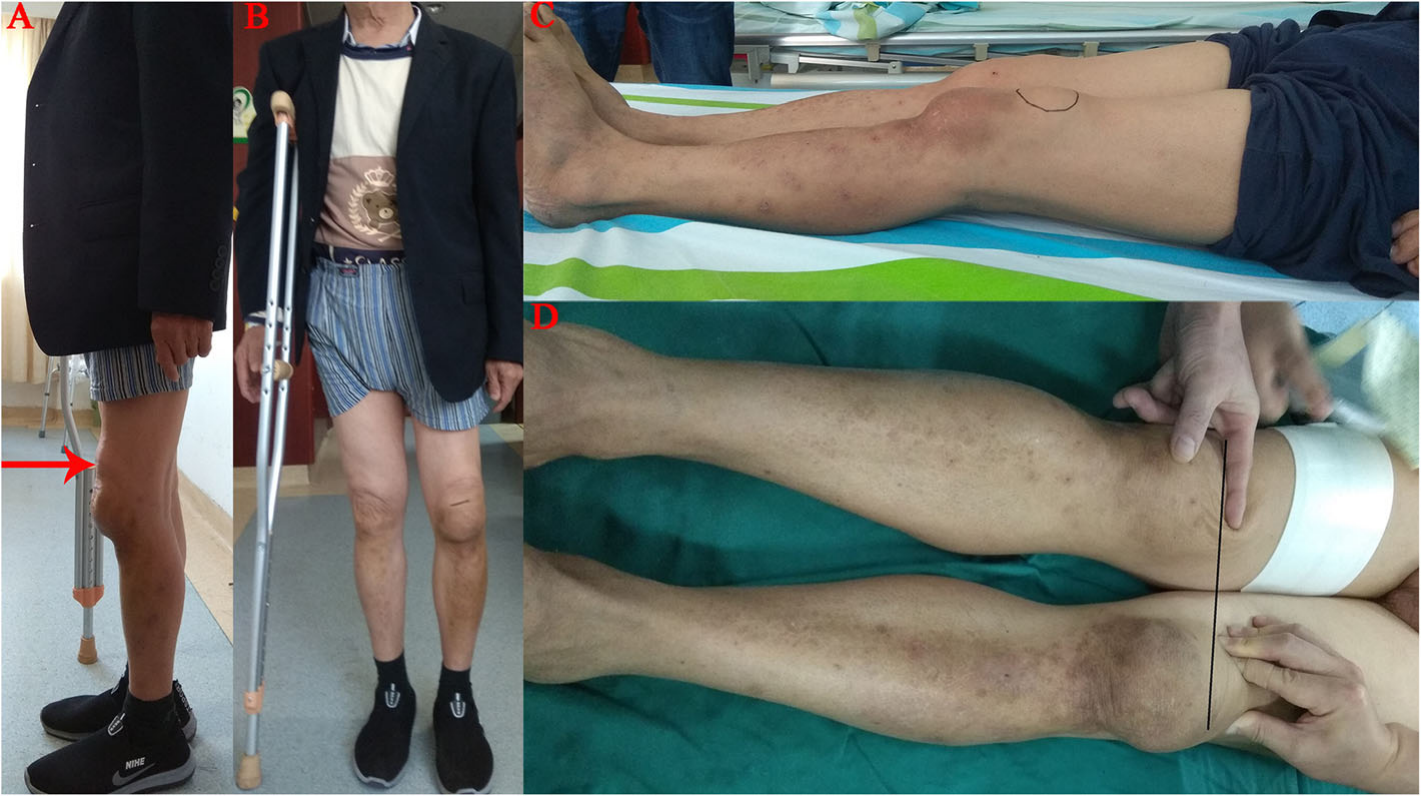
Preoperative overall photo. **a** Load-bearing lateral position, the red arrow indicates upward displacement of the patella, patella alta; **b** Load-bearing positive position; **c** Non-load lateral position; **d** Non-load positive position, the patella can be pushed down

**Fig. 2:**
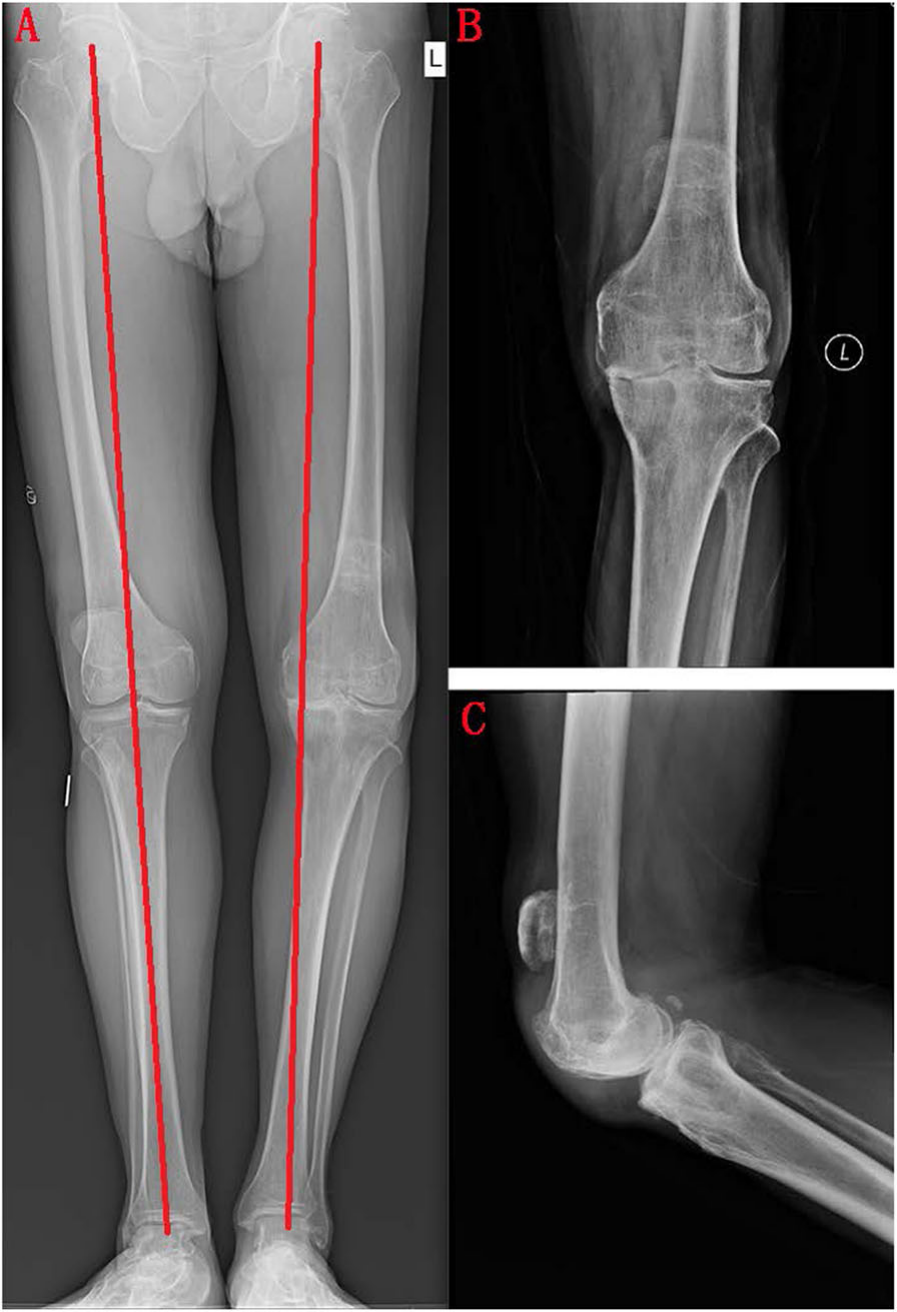
Preoperative X-ray. We found advanced degenerative arthritis of the left knee; the knee joint had mild varus deformity according to the full-length film of the lower limbs and positive and lateral radiographs of the knee joint

**Fig. 3:**
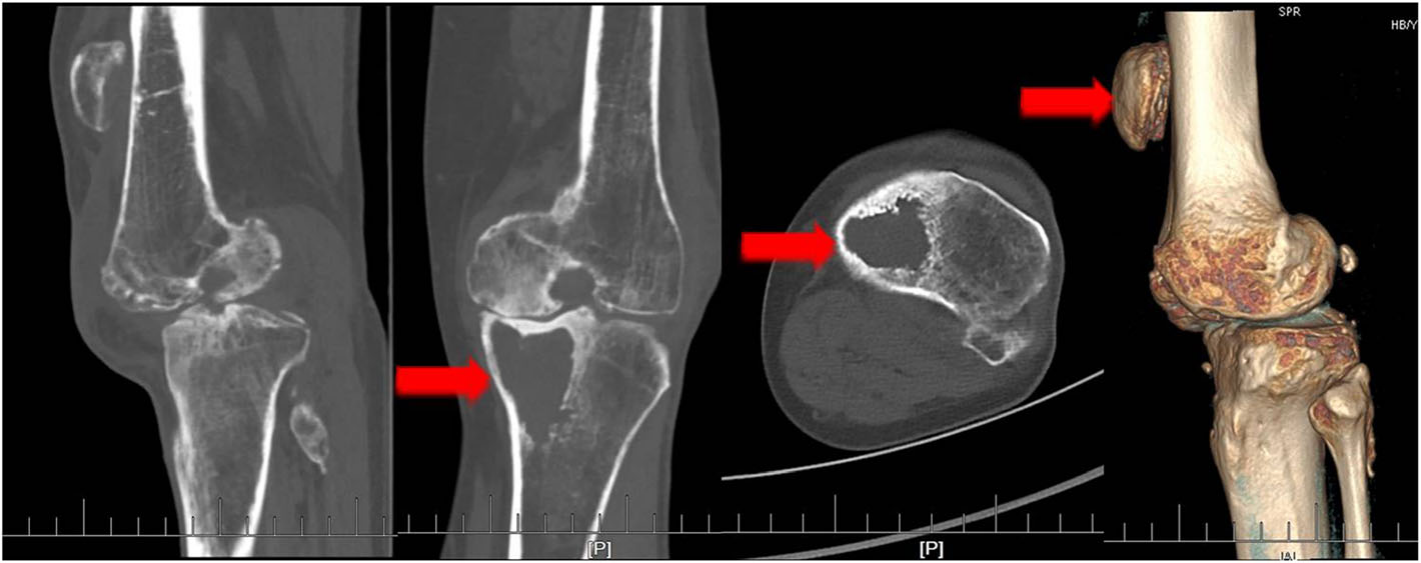
Preoperative 3D-CT. The left and middle arrows show cystic changes under the medial tibia of the left knee joint. The right arrow indicates patella alta. The Insall-Salvati ratio was 1:3 from preoperative 3D-CT. Compared with the other side, we believe that the height of the left patella is consistent with that of the patella alta

A 67-year-old male patient reported that he suffered from patellar ligament rupture due to an accidental fall more than 20 years ago, and he did not receive any formal treatment. Later, the knee strength gradually decreased, and left quadriceps muscle atrophy appeared. The patient developed pain and swelling in the left knee 6 months ago, and needed a walking stick to assist walking (supplemental video 1). The VAS score was 5 points before surgery. The patient described that he could not tolerate the pain caused by walking. Three months ago, joint cavity paracentesis was performed to extract joint fluid to reduce joint swelling. However, the swelling of the joint recurred after a week. Physical examination revealed a small amount of fluid in the left knee joint, and the patella was displaced upward by approximately 5.5 cm (Fig. 1). The quadriceps were atrophic and weak. There was significant tenderness on the medial side of the left knee joint. Passive motion of the left knee joint ranged from full extension to 120° of flexion with discomfort during excessive flexion. Active flexion of the knee joint to 120°, and extensor lag was approximately 90°. The patient could not actively straighten the knee. We did not find any significant knee instability or observe patella tracking when flexing and straightening the knee joint. The Lachman, McMurray, and anteroposterior drawer tests were negative. There was grade 0 strength during extension of the left knee, and grade V strength during extension of the right knee. Before the operation, we routinely performed tests for biochemical indicators such as routine blood, C-reactive protein (CRP, 2.4 mg/L, normal, < 5 mg/L), erythrocyte sedimentation rate (ESR, 10 mm/h, normal, < 43 mm/h), procalcitonin (PCT, 0.12, normal, < 0.5) and other biochemical indicator tests to exclude knee infection. Full-length films of the lower limbs, three-dimensional computed tomography (3D-CT) of the knee joint, and positive and lateral radiographs of the knee joint were also performed before the operation. X-ray photographs showed advanced degenerative arthritis of the left knee with significant involvement of the medial compartment but less significant lateral compartment changes, and the knee joint had mild varus deformity. We could see patella alta in this image (Fig. 2). 3D-CT showed cystic changes under the medial tibia of the left knee joint (Fig. 3). According to the typical left knee pain and disability of the patient, we offered three options: TKA and reconstruction of the patellar ligament, nonoperative treatment, and knee fusion. The patient strongly demanded the first option. Informed consent was obtained from the patient.

**Fig. 4:**
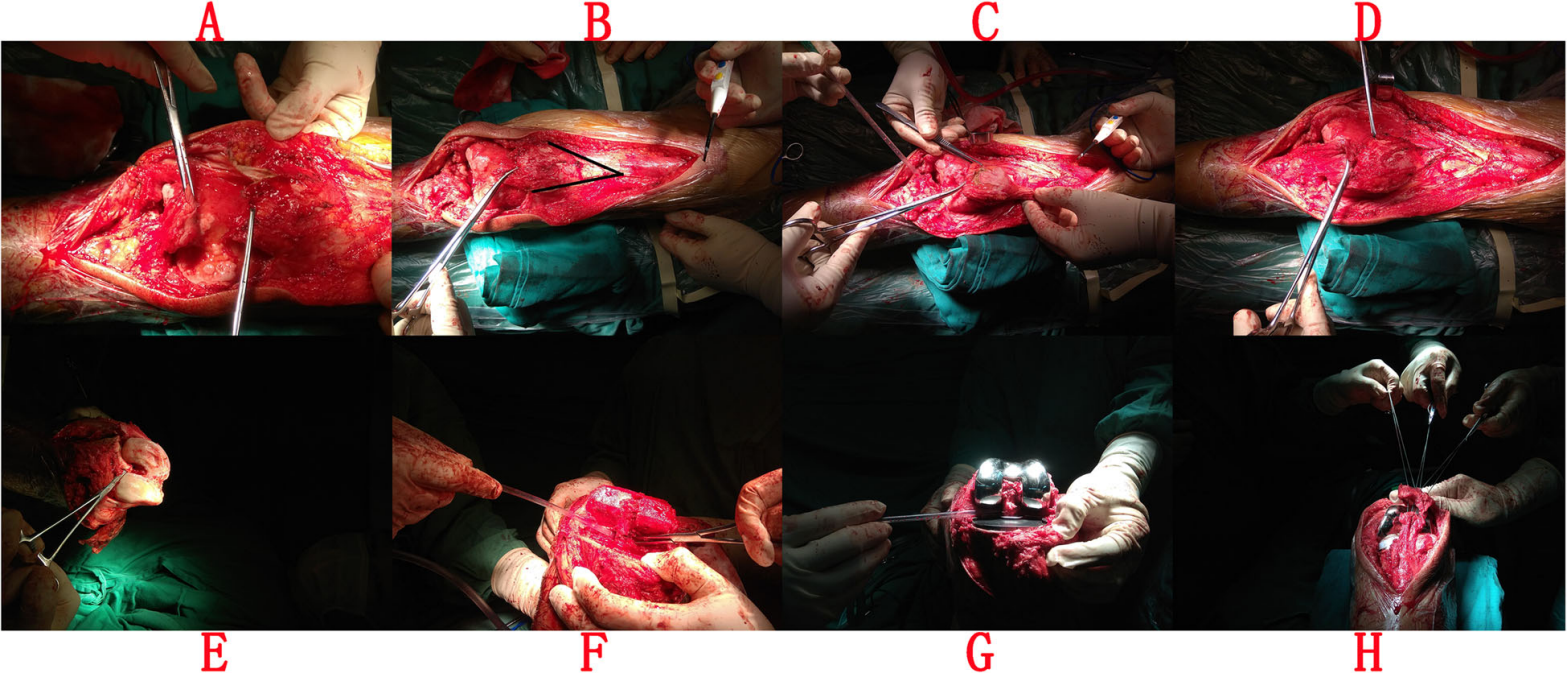
**a** Chronic patellar ligament rupture, the patellar ligament ruptured approximately 1.5 cm below the inferior patellar pole, and the termination of the patellar ligament on the tibial tubercle was complete. The patella can be pushed down approximately 4 cm. The ruptured tendon was flocculent, with irregular broken ends and atrophy of patellar ligament. **b** Preparing for quadriceps tendon V-Y quadricepsplasty. **c** Patellar displacement distance was measured after patellar ligament connected to patella, then decided the length of quadriceps snip. **d** After V-Y lengthening of quadriceps femoris tendon. **e** After opening the capsule articularis genus, we found synovial hyperplasia, degeneration of the medial and lateral menisci, osteophyte formation around the joint, articular cartilage degeneration and wear, and subchondral bone exposure, especially of the medial compartment. We preserved posterior cruciate ligament. **f** After distal femur and proximal tibia osteotomy was completed. We preserved posterior cruciate ligament. **g** Installing the prosthesis, bone cement filled bone defect. **h** The locking suture knots were placed into the patellar tendon with nonabsorbable sutures. The two ends of the same suture pass through adjacent tunnels by Krakow suture technique. The suture was tied over the superior pole of the patella

**Fig. 5:**
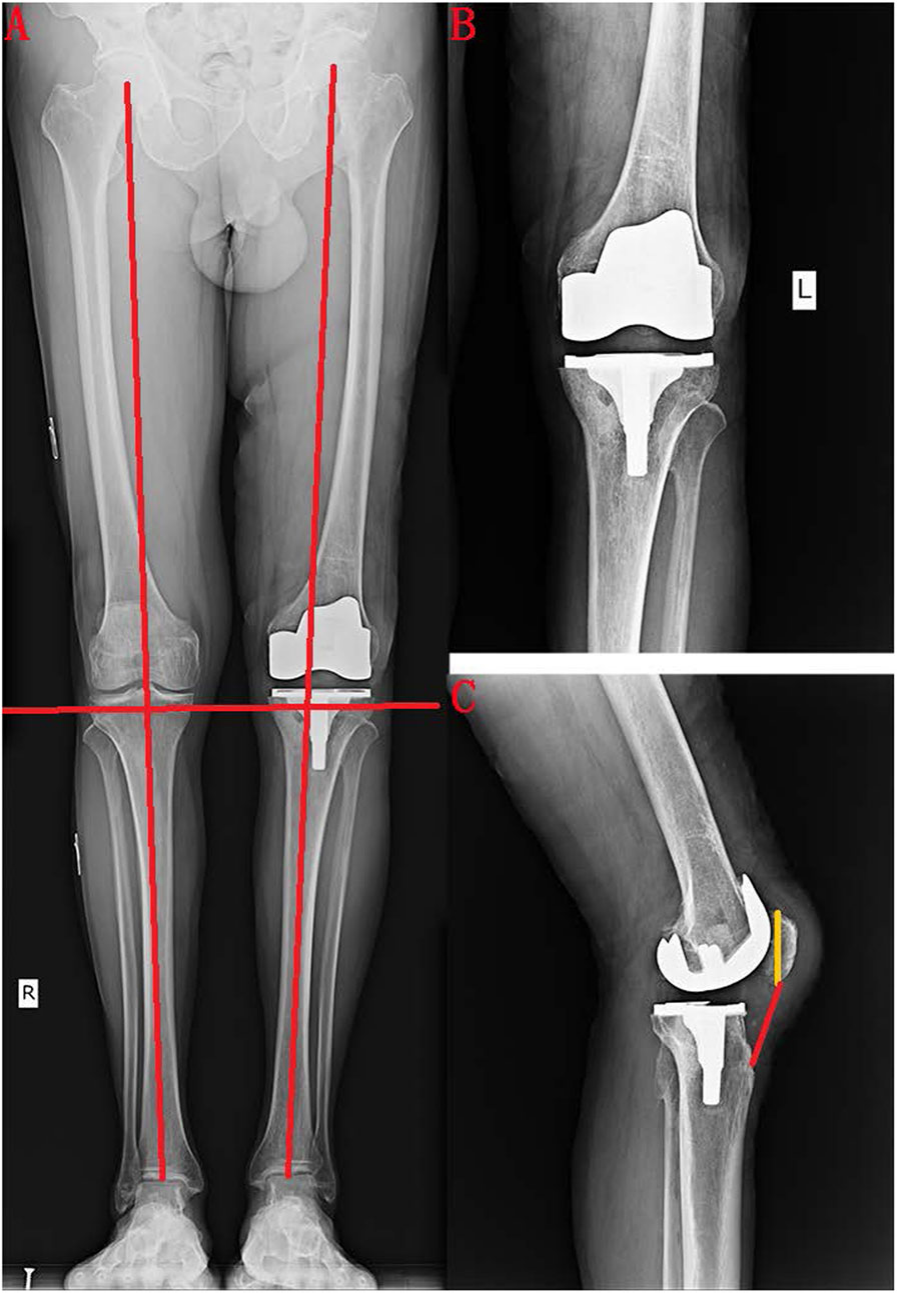
Postoperative full-length film of the lower limbs and positive and lateral radiographs of the knee joint. The varus deformity of the left lower extremity was corrected, and the joint lines of both knees were at the same level. The Insall-Salvati ratio was 0.85. The Insall-Salvati ratio (ratio of the length of the patellar tendon (measured from the distal pole of the patella to the tibial tuberosity) to the maximum length of the patella (measured from the distal pole to the proximal pole of the patella)) was between 0.72 and 0.9 under average conditions

We chose the GENESIS^◊^ II Cruciate Retaining Total Knee Replacement prosthesis (Smith & Nephew, Memphis, Tennessee, USA) to perform this surgery. Total knee replacement was performed with a medial parapatellar approach. When we opened the knee capsule, we found more joint fluid in the joint cavity, synovial hyperplasia, degeneration of the medial and lateral menisci, osteophyte formation around the joint, articular cartilage degeneration and wear, and subchondral bone exposure, especially of the medial compartment (Figs. 4e and 5). We found that the patellar ligament was ruptured about 1.5 cm below the inferior patellar pole, and the termination of the patellar ligament on the tibial tubercle was complete. The ruptured tendon was flocculent, with irregular broken ends and atrophy of the patellar ligament (Fig. 4a). The patella was displaced upward approximately 5.5 cm, consistent with preoperative examinations. The distance of patellar displacement was measured after the connection of the patellar ligament to the patella, and then the length of the quadriceps snip was determined (Fig. 4a, b). We performed osteotomy according to the joint prosthesis instructions and installed the prosthesis (Fig. 4f, g). We chose femur (5#) and tibia (3–4#) prosthese and a sacral gasket test pattern (3–4#, 11 mm). After assembling the components, the articular surface of the patella was resurfaced. Finally, we performed a V-Y quadricepsplasty to lengthen the quadriceps tendon (Fig. 4b-d). We drilled four parallel bone tunnels approximately 2.5 mm in diameter under the patella. The locking suture knots were placed into the patellar tendon with nonabsorbable sutures. The two ends of the same suture were passed through adjacent tunnels via the Krakow suture technique. The suture was tied over the superior pole of the patella (Fig. 4h) [6]. We used a knee external fixator to fix the knee joint in a 30° flexion position after surgery.

After 1 week, he started knee function exercises from 0° to 30° with a joint loosening trainer and walked with the help of crutches. The patient’s VAS score decreased from 5 points before surgery to 1 point after surgery. One week later, the range of joint motion increased to 90° (supplemental video 2). X-ray examination showed no obvious unequal lengths of the lower limbs or varus or valgus deformity after 1 week. Ten months after the operation, the active ROM was 0°-30°-120°, the passive ROM was 0°-10°-120°, and the extension lag was improved. The knee strength during extension of the left knee increased to grade IV. We will further track the postoperative recovery of the patient.


**Additional file 2: Supplement video 2.** One week after surgery, the range of joint motion increased to 90°.


## Discussion and conclusions

TKA is an effective and well-developed surgical procedure for the treatment of severe osteoarthritis. Extensor apparatus rupture is a rare but severe complication after TKA, resulting in insufficient active knee extension, which can seriously impair knee function [7]. However, for OA patients with chronic patellar ligament rupture, how do we make a surgical plan? Can we choose TKA and perform ligament reconstruction at the same time? How to reconstruct a fractured patellar ligament is a considerable challenge for patients after TKA. It is highly difficult to repair the chronic rupture of the patellar ligament. First, patellar ligament rupture mainly occurrs in middle-aged individuals and is associated with direct traumatic mechanisms or end-stage patellar tendinopathy. Patellar tendon rupture is thought to be the culminating end-stage event of chronic tendon degeneration secondary to repetitive microtrauma. Second, after the patellar ligament is broken, the patella is displaced upwards under traction of the quadriceps tendon, leading to dislocation of the patella. In chronic tendon ruptures, retraction of the quadriceps severely compromises the feasibility of direct repair. In this review, the authors summarized several surgical techniques for reconstruction of the fractured patellar ligament [3]. These choices include simple repair or repair with augmentation by a neighbouring tendon, by an artificial ligament or by an allograft. Another option is the use of salvage techniques, which create a gastrocnemius flap or vastus medialis and/or lateralis flap that provides vascularized tissue for attachment to the remnants of the extensor mechanism. In the final stage, we can also choose knee arthrodesis. The choice of surgical method depends on many factors, such as the site and duration of the rupture, partial or complete tears, a history of surgery and/or infection, the patient’s general health status, the degree of discomfort, the functional demands, and the availability of allografts [3]. In this case, according to his age and the duration of rupture, we recommended that the patient choose knee arthrodesis, but he refused this treatment, and requested retaining the range of motion of the knee joint.

Dennis reported a method to repair quadriceps and patellar tendon ruptures [6]. In this study, he showed that the basic principle of reconstruction of the patellar ligament was that end-to-end suturing repaired the patellar ligament, but they did not recommend this method because of the high rate of re-rupture. They also detailed how to reconstruct the patellar ligament with transpatellar tunnels. They drilled three parallel bone tunnels approximately 2.5 mm in diameter under the patella, and nonabsorbable sutures were then placed in the patellar ligament using a Krakow or equivalent suture technique; the ends of the sutures were passed through the tunnels and tied down. This method was proven to be an effective method for the reconstruction of simple patellar tendon injuries. However, we did not find any experience with simultaneous TKA and patellar ligament reconstruction. Therefore, in our study, we evaluated how to perform patellar tendon repair while performing TKA. In addition, V-Y quadricepsplasty has been widely used in severe extensor mechanism contracture during TKA [8, 9]. V-Y quadricepsplasty is much safer than osteotomy of the tibial tubercle when performing TKA. In cases of revision or stiff knees, V-Y quadricepsplasty provides good surgical exposure. However, this procedure results in extension lag (from 8° to 27°) and flexion contractures, mild to moderate pain, and weakness in the knee extensor muscles [10]. V-Y quadricepsplasty may also affect walking distance and both going up and down stairs. However, this degree of knee extension lag is perfectly acceptable and has little effect on daily activities [8, 10]. Although both V-Y quadricepsplasty and reconstruction of the patellar ligament were not our original focus, we did not find case reports of the combined use of V-Y quadricepsplasty, TKA and patellar ligament reconstruction. In our case, we believe that we chose the best treatment for our patient.

Before surgery, we found that the patella could move downward, and the distance of the patella was reduced by about 4 cm (Fig. 1d). During the operation, we also found little retraction of the quadriceps tendon, but it did not affect our patellar reduction (Fig. 1a). In any case, direct repair was the first choice, although simple suture has a high probability of failure [3]. Therefore, we performed quadriceps tendon V-Y quadricepsplasty to restore the patellar tendon (Fig. 1b) and then chose a simple suturing ligament with the Krackow suture technique [11], and drilled under the inferior pole of the patella to form 4 bone tunnels and cross-stitching. After we finished V-Y tendon extension of the quadriceps femoris, TKA was performed with a cruciate-retaining total knee replacement prosthesis. Compared with cruciate-retaining (CR) TKA, cruciate-sacrificed TKA is less efficient and has more medial loading and higher joint reaction forces that may affect the durability of the prosthesis [12]. Posterior cruciate ligament (PCL)-retaining TKA has a more normal and efficient gait pattern [12]. Mechanoreceptors are embedded in the posterior cruciate ligament, so retaining the posterior cruciate ligament may result in superior proprioceptive activity after TKA. PCL-retaining maintains femoral rollback and increases the dynamic quadriceps lever arm, which is responsible for improved strength [13]. However, a recent study reported that there was no difference in terms of proprioception and muscle strength between the PCL-retaining and PCL-resected groups [14]. Retaining the posterior cruciate ligament prosthesis retains more bone mass and facilitates revision surgery. Considering the patient’s preoperative gait pattern and knee joint range of motion (ROM), we chose a CR total knee replacement prosthesis. Six weeks later, the patient was able to walk normally without a walking stick (supplemental video 3), and the knee joint could stretch actively approximately 30° (supplemental video 4). Ten months after the operation, the active ROM was 0°-30°-120°, the passive ROM was 0°-10°-120°, and the extension lag was improved. The knee strength during extension of the left knee increased to grade IV (supplemental videos 5 and 6). The patient could go up and down stairs without crutches, and walk at least 2000 m along a level floor. Before surgery, the AKS score was 35, but it increased to 95 10 months after surgery. The HSS scores increased from 43 to 93. The patient is very delighted with the result of the operation.


**Additional file 4: Supplement video 4.** Six weeks after surgery, the knee joint can stretch actively about 30°. But this patient does have extension lag.


Knee OA with chronic patellar ligament rupture is a rare disease. TKA and ligament reconstruction are options for the treatment of this condition. V-Y lengthening of the quadriceps femoris tendon after the Krackow suture technique of the patellar ligament with transpatellar tunnels may be the right choice for this person. We have demonstrated reasonable short-term success, fulfilling the patient expectations but with residual functional limitations regarding active extension. Although re-rupture is possible, we will follow the patient in terms of outcomes.

## Supplementary information

**Additional file 1 Supplement video 1.** Preoperative gait, he needed a walking stick to assist walking.

**Additional file 3 Supplement video 3.** Six weeks after surgery, the patient was able to walk normally without the walking stick.

**Additional file 5 Supplement video 5.** Ten months after the operation, he is able to walk normally without use of crutches. Ten months after the operation, the active ROM was 0°-30°-120° and passive ROM is 0°-10°-120°, and the extension lag was obviously improved. The knee strength during extension of the left knee increased to grade IV.

**Additional file 6 Supplement video 6** Ten months after the operation, he is able to walk normally without use of crutches. Ten months after the operation, the active ROM was 0°-30°-120° and passive ROM is 0°-10°-120°, and the extension lag was obviously improved. The knee strength during extension of the left knee increased to grade IV.

## Data Availability

Not applicable.

## Abbreviations

3D-CT: Three-dimensional Computed Tomography
CRP: C-reactive protein
CR: Cruciate retaining
ESR: Erythrocyte sedimentation rate
Fig.: Figure
OA: Osteoarthritis
PCT: Procalcitonin
PCL: Posterior cruciate ligament
ROM: Range of motion
TKA: Total knee arthroplasty
VAS: Visual analog scale
